# Gut microbiota and its complex role. The experience of the National Institute for Infectious Diseases “Prof. Dr. Matei Balş” in fecal bacteriotherapy for *Clostridium difficile* infection

**DOI:** 10.1186/1471-2334-13-S1-O19

**Published:** 2013-12-16

**Authors:** Cătălin Apostolescu, Ruxandra Moroti, Violeta Molagic, Valeriu Gheorghiță, Daniela Tălăpan, Mona Popoiu, Smaranda Botea, Alexandru Rafila, Marilena Palaghiță, Anca Budulac, Lavinia Ivănescu, Mirela Trifan, Gheorghiță Ciobanu, Adrian Streinu-Cercel

**Affiliations:** 1 National Institute for Infectious Diseases “Prof. Dr. Matei Balş”, Bucharest, Romania; 2 Carol Davila University of Medicine and Pharmacy, Bucharest, Romania; 3 ”Dr. Carol Davila” Central Military Emergency University Hospital, Bucharest, Romania

## Background

The human microbiome, meaning the total group of microbes that constantly populates a human being, has been intensively studied in the lasts years. The gut microbiota seems to be the most important part of it, not just regarding the number of cells and diversity, but for the almost unbelievable role in maintaining health, acting on the immunity-disease axis. The gut can be interpreted as a “microbial organ” and one obvious aspect of its deterioration, mainly as result of antibiotics misuse, is *Clostridium difficile* colitis.

In order to reverse the altered gut flora, a team consisting of clinicians and microbiologists developed a protocol, in accordance with internationals protocols, with specific procedures, for performing fecal bacteriotherapy in patients with recurrences of *Clostridium difficile* infection. Fecal bacteriotherapy consists in transplantation of a small quantity of feces from a healthy donor to another person with abnormal intestinal flora.

## Case report

Two female patients aged 80 respectively 83 years old received fecal bacteriotherapy in our Institute, for the second, respectively for the third *Clostridium difficile* recurrence (ATLAS score <5); one case received the fecal transplant by enema and one by nasogastric route. The procedure was well tolerated. The evolution was favorable with rapid remission of diarrhea in the next 48 hours and with no relapses in the 30 days of follow-up.

## Conclusion

It is necessary to extend the experience with this treatment, the fecal bacteriotherapy being a viable alternative for *Clostridium difficile* infections failure to standard treatment.

